# Gut and Vaginal Microbiota in the Endometriosis: Systematic Review and Meta-Analysis

**DOI:** 10.1155/2023/2675966

**Published:** 2023-05-29

**Authors:** Tamy Colonetti, Maria Carolina Saggioratto, Antonio José Grande, Laura Colonetti, João Carlos Denoni Junior, Luciane Bisognin Ceretta, Leonardo Roever, Fábio Rosa Silva, Maria Inês da Rosa

**Affiliations:** ^1^Laboratory of Biomedicine Translational, Universidade do Extremo Sul Catarinense, Av. Universitária, 1105-Bairro Universitário CEP, 88806-000 Criciúma, SC, Brazil; ^2^Laboratory of Evidence-Based Practice, Universidade Estadual de Mato Grosso do Sul (UEMS), Campo Grande, Mato Grosso do Sul, Brazil; ^3^Postgraduate Program in Collective Health, Universidade do Extremo Sul Catarinense (UNESC), Criciúma, Santa Catarina, Brazil; ^4^Department of Clinical Research, Federal University of Uberlândia, Uberlândia, Brazil

## Abstract

**Background:**

Endometriosis is a clinical condition associated with genetic, endocrine, and immunological factors, present in 6 to 10% of women of reproductive age. Currently, the human microbiota has been studied and associated with the evolution of diseases due to its influence on pathogenesis, indicating that changes in the colonization of microorganisms in the genitourinary and gastrointestinal systems can promote physiological changes that can trigger inflammatory and immunological processes and hormonal dysregulation, which can be linked to endometriosis. Thus, this systematic review and meta-analysis evaluated microbiota changes in women with endometriosis.

**Methods:**

The following electronic databases were searched up to April 2022: Medline, Embase, Web of Science, Cochrane Library, and gray literature (Google Scholar), using the keywords “dysbiosis”, “microbiome” and “endometriosis”, combined with their synonyms. The observational studies conducted with women diagnosed with endometriosis and women without endometriosis as controls were included. For the analyses, a standard mean difference with a 95% confidence interval was used using RevMan software (version 5.4), and for methodological quality assessment, the Newcastle-Ottawa scale was used.

**Results:**

A total of 16 studies were found in the literature assessing the composition of the microbiota in women with endometriosis, and no significant difference were found for changes in alpha diversity analysis in gut microbiota (SMD = −0.28; 95% CI = −0.70 to 0.14; *P* = 0.19; *I*^2^ = 52%; four studies, 357 participants) or vaginal microbiota (SMD = −0.68; 95% CI = −1.72 to 0.35; *P* = 0.19; *I*^2^ = 66%; two studies, 49 participants).

**Conclusion:**

In intestinal and vaginal samples from women with endometriosis, alpha-diversity did not present a significant difference when compared to the control population. However, each study individually showed a possible relationship between the female microbiota and endometriosis. This trial is registered with CRD42021260972.

## 1. Background

Endometriosis is characterized by the growth of endometrial tissue in extrauterine sites, such as the visceral and peritoneal surfaces of the pelvis and in the connective tissue of the extrpelvic region [1, 2]. Endometriotic lesions have endometrial glands and stroma and are accompanied by cyclic and recurrent bleeding, progressive formation of fibrosis and cysts, producing chronic inflammation with increased angiogenesis, and altered immune functions [3].

Recent evidence has shown that changes in the intestinal microbiota, known as dysbiosis, may lead to the development and progression of several diseases, such as inflammatory bowel diseases, arthritis, psoriasis, neuropsychiatric diseases, and even some types of cancer [4–6]. These diseases are related to the inflammatory process, which is also present in endometriosis. Thus, studies investigating dysbiosis in the genital tract or pelvic cavity may be associated with the pathogenesis and/or pathophysiology of endometriosis [7].

Microbiota provides several benefits through a series of physiological functions, acting to strengthen the integrity of the intestines [8], protect against diseases [9], and also for the regulation of the immunological system [10]. However, changes in its composition can generate an imbalance in the microbiota (dysbiosis), which could predispose to the emergence of diseases and metabolic dysfunctions as the protection against microorganisms decreases [11, 12].

Furthermore, the microbiota is also involved in adjustments to the estrogen cycle. Studies have shown that intestinal dysbiosis is a factor in increasing the levels of estrogen in the circulation, thus being able to stimulate the growth and cyclic bleeding of endometriotic lesions [13]. Therefore, this study maps the evidence in the literature regarding the association between intestinal and vaginal microbiota in endometriosis.

## 2. Materials and Methods

The study is characterized as a systematic review of the literature, written according to the PRISMA checklist (preferred reporting items for systematic reviews and meta-analyses) [14]. Registration was carried out in PROSPERO (international prospective register of ongoing systematic reviews, http://www.crd.york.ac.uk/prospero) CRD42021260972, 15/07/2021.

### 2.1. POS

POS stands for an acronym for patient, outcomes, study type [15], in which each letter represents an organized summary of the inclusion criteria presented below:

P. Population of interest: women over 18 years old with and without endometriosis

O. Outcomes (outcome): changes in intestinal, vaginal, and cervical microbiota

S. Study type: observational studies (case-control, cross-sectional, or cohort)

### 2.2. Search Strategy

A comprehensive electronic search was conducted on Medline via PubMed, Embase via Elsevier, Web of Science through Web of Knowledge, Cochrane Library, and gray literature (Google Scholar) databases up to April 2022. The search strategy was organized using the following keywords: “dysbiosis,” “microbiome,” and “endometriosis” in combination with their respective synonyms, indexed terms, and free terms. Reference lists of all available primary studies were reviewed to identify additional relevant citations. The search was limited to human studies, with no language restrictions.

### 2.3. Selection of Studies

Two reviewers (MCS and TC) independently screened the titles and abstracts through the Rayyan application (https://www.rayyan.ai) [16]. The potentially relevant studies were read in full, and those that met the eligibility criteria were included in this review. When there was any disagreement, a third reviewer was contacted to resolve the conflict (MIR).

### 2.4. Inclusion and Exclusion Criteria

Studies with women over 18 years old, women with a histologically confirmed diagnosis of endometriosis, and women without endometriosis, as controls, were included. Studies with women with current inflammatory comorbidities that could alter the flora or those using antibiotics were excluded.

### 2.5. Microbiota Assessment

DNA was extracted from the intestinal microbiota through stool samples collected according to the instructions of the kits used in each study. DNA was extracted from vaginal and cervical microbiota samples by swabbing, by a qualified professional. Each type of sample was processed and stored according to directions from the manufacturers of the kits used in each included study.

In all samples, the 16S rRNA genomic extraction methodology was used, which is based on the detection of sequences from the highly variable region of the 16S rRNA gene, in order to identify the microbial composition and richness, that is, their taxonomic profile.

In the studies included, richness was evaluated through alpha diversity, which also considers the proportion in which these species occur in each habitat (relative abundance) [17].

### 2.6. Data Extraction

Two reviewers (MCS and TC), independently, extracted data using a standard form containing author, year and country of the study, age group, endometriosis diagnostic method, endometriosis staging, type of sample analyzed: intestinal, cervical, vaginal, or other, and index used to analyze the diversity and composition of the microbiota. In order to extract data from the figures, the website https://apps.automeris.io/wpd/ was used.

### 2.7. Risk of Bias Assessment

Two reviewers (MCS and TC), independently, assessed the methodological quality (risk of bias) of the studies using the Newcastle-Ottawa scale.

### 2.8. Data Analysis

The results were expressed through tables and figures. Forest plots were created to illustrate the effect sizes studied for certain outcomes. For the analyses, a 95% confidence interval was used using RevMan software (version 5.4). Study heterogeneity was determined using the *I*^2^ statistic, from 0% to 40%: may not be important; 30% to 60%: may represent moderate heterogeneity; 50% to 90%: may represent substantial heterogeneity; 75% to 100%: considerable heterogeneity [18]. When heterogeneity was suspected, random-effect model estimates were used. When no heterogeneity was observed, the Mantel-Haesnzel fixed-effect model was used.

## 3. Results

**Figure 1:**
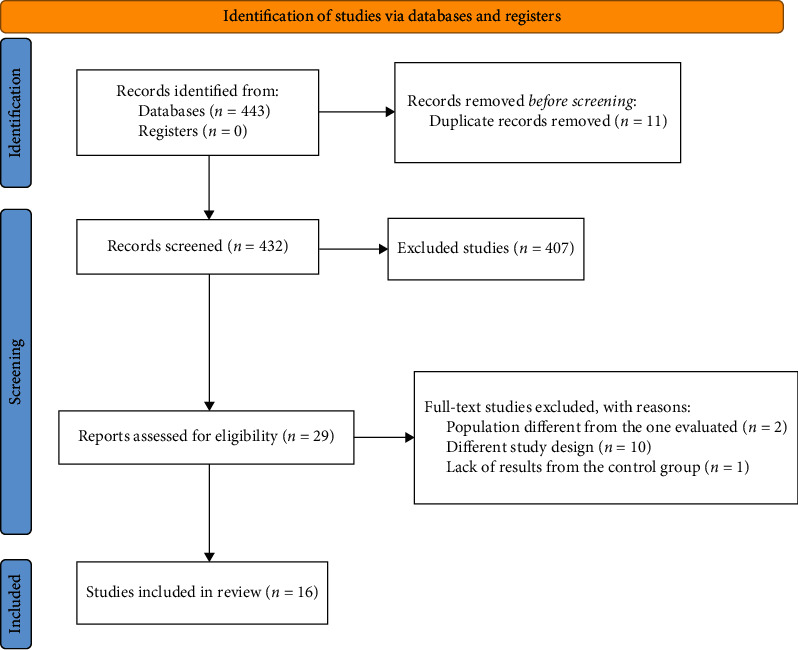
PRISMA Flow-diagram for study selection.

A total of 443 studies were found across databases, and 11 studies were removed as duplicates. Thus, 432 studies were screened by reading titles and abstracts in Rayyan, and a total of 407 studies were excluded for not meeting the inclusion criteria. A total of 29 studies were selected for full text reading; 13 were excluded for the following reasons: two studies due to the wrong population including infertile women and not only due to endometriosis; ten studies due to study design: four literature reviews, two letters/comments to the editor, and four intervention studies; and one study for not presenting data from the results of the control group. Thus, a total of 16 studies were included in this systematic review.  Figure 1 presents the flowchart of the study process.

One cohort study [19], one cross-sectional study [20], and fourteen case-control studies were included [21–34], totaling 1151 women studied, of which 556 had endometriosis and 595 were part of control groups. Endometriosis diagnosis was performed through laparoscopy and stage classification according to the American Society of Reproductive Medicine (r-ASRM) [35]. The characteristics of the included studies are described in Table 1.

### 3.1. Microbiota Analysis-Outcomes

#### 3.1.1. Alpha Diversity Assessed by Shannon Index

Ten studies [19, 21, 22, 25, 27, 28, 31–34] performed the analysis of alpha diversity using the Shannon index, which relates the richness of operational taxonomic units (OTU) and equity by the total number of species observed. A meta-analysis was performed for the intestinal and vaginal microbiota. Samples of cervical, endometrial microbiota, and peritoneal fluid were evaluated but showed heterogeneity greater than 80%; thus, the data regarding alpha diversity from these studies are available in Table 2.

**Figure 2:**
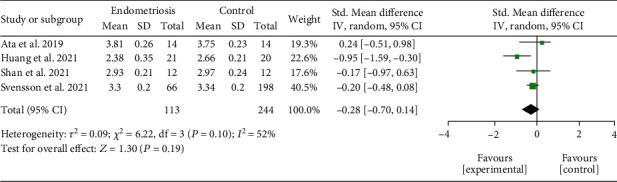
Meta-analysis for the comparison between women with endometriosis and women in control groups for intestinal alpha diversity assessed by Shannon index. Figure legend: SD: standard deviation; CI: confidence interval.

*(1) Intestinal Alpha Diversity.* Four included studies [19, 27, 28, 32] performed alpha diversity analysis of intestinal microbiota. No significant differences were found when comparing women in the endometriosis group with the control group for changes in the intestinal microbiota (SMD = −0.28; 95% CI = −0.70 to 0.14; *P* = 0.19; *I*^2^ = 52%; four studies, 357 participants). The results of intestinal alpha diversity by Shannon index meta-analysis are represented in Figure 2.

**Figure 3:**
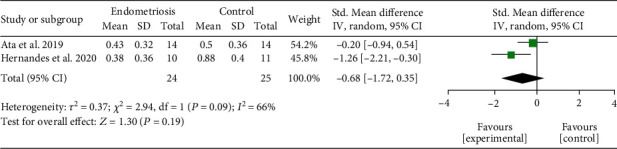
Meta-analysis for the comparison between women with endometriosis and women in control groups for vaginal alpha diversity assessed by Shannon index. Figure legend: SD: standard deviation; CI: confidence interval.

*(2) Vaginal Alpha Diversity.* Two studies analyzed the vaginal microbiota [19, 22]. When comparing the richness of diversity, no significant difference was found between microbiota samples from women with endometriosis and women from the control groups (SMD = −0.68; 95% CI = −1.72 to 0.35; *P* = 0.19; *I*^2^ = 66%; two studies, 49 participants). The results of vaginal alpha diversity by Shannon index meta-analysis are represented in Figure 3.

#### 3.1.2. Alpha Diversity Assessed by Simpson Index

Five studies [22, 25, 27, 32, 34] performed the analysis of alpha diversity using the Simpson index, evaluated to measure community diversity in order to represent the proportion of species in a sample, for example, the dominance of one species among others, and to measure differences in diversity between populations. A meta-analysis was performed for intestinal and peritoneal fluid.

**Figure 4:**
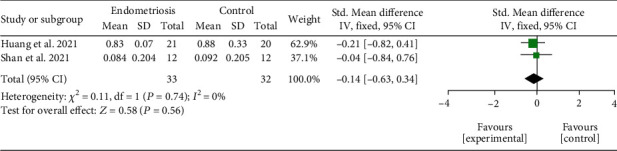
Meta-analysis for the comparison between women with endometriosis and women in control groups for intestinal alpha diversity assessed by Simpson index. Figure legend: SD: standard deviation; CI: confidence interval.

*(1) Intestinal Alpha Diversity.* Two studies [27, 32] analyzed the intestinal microbiota. When comparing endometriosis and the control group, no significant difference was found between microbiota samples (SMD = −0.14; 95% CI = −0.63 to 0.34; *P* = 0.56; *I*^2^ = 0%; two studies, 65 participants). The results of intestinal alpha diversity by Simpson index meta-analysis are represented in Figure 4.

**Figure 5:**
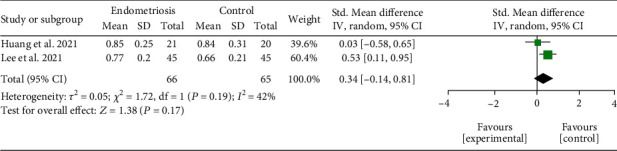
Meta-analysis for the comparison between women with endometriosis and women in control groups for peritoneal fluid alpha diversity assessed by Simpson index. Figure legend: SD: standard deviation; CI: confidence interval.

*(2) Peritoneal Fluid Diversity.* Peritoneal fluid analysis was evaluated in three studies [25, 32, 34], but data from Yuan et al., [34] were in the median and confidence interval. Thus, two studies [25, 32] were included in the analysis. Although the groups with endometriosis showed higher values, no significant difference was found between microbiota samples from peritoneal fluid (SMD = 0.34; 95% CI = −0143 to 0.81; *P* = 0.17; *I*^2^ = 42%; two studies, 131 participants). The results of peritoneal fluid diversity by Simpson index meta-analysis are represented in Figure 5.

The considerable heterogeneity found between the studies can be attributed to different factors that change the characteristics of the microbiota, such as diet, use of medication (especially antibiotics), country of residence of the studied population, cultural factors, as well as lifestyle habits.

### 3.2. Composition of Microbiota

Due to the wide variety of presentations of the results of the microbiota compositions, we were not able to perform a quantitative analysis. Thus, the main results found in studies regarding the composition of the microbiota are presented in a descriptive way. Information regarding the methodological aspects and results is described in Table 3.

### 3.3. Analysis of the Methodological Quality of the Included Studies

The methodological quality assessment of the included studies was performed using the Newcastle-Ottawa scale (NOS). NOS were used to assess a cohort study (one study), case-control studies (14 studies), and a cross-sectional study (one study). For case-control studies, in all studies, a quality mean of 8/9 was obtained. We consider studies with 7 stars or higher as high quality and have a low risk of bias. For the 6/9 cohort study, high risk of bias, and for the cross-sectional study, the quality score was 7/9, a high quality and low risk of bias.  Table 4 shows the results of the methodological quality analysis.

## 4. Discussion

### 4.1. Principal Findings

In this systematic review, four meta-analyses were carried out regarding alpha diversity assessed by Shannon and Simpson indexes in the intestinal, vaginal, and peritoneal fluid samples, with no significant association. However, when analyzing each study individually, differences were noted between the microbiota of women with endometriosis compared to the microbiota of women in control groups. This occurred in the analysis of intestinal and vaginal samples. Simpson's index represents the proportion of species in a sample, measuring the diversity of the community. The range is from 0 to 1, where high scores indicate high diversity. Analyzing the studies included in the analysis of the intestinal sample, the control population has greater diversity by the Simpson index, while in the peritoneal fluid, this diversity is greater in the population with endometriosis, although showed no significant difference, possibly due to the limited number of studies.

### 4.2. Comparison with Existing Literature

The intestinal dysbiosis process may be related to the pathogenesis of endometriosis, as it compromises the barrier function, causing increased permeability and entry of microbial metabolites, which may trigger inflammatory changes, leading to acute inflammation in places outside the gastrointestinal tract, such as the peritoneum [36].

This change could result in low-grade inflammation and, over time, lead to changes in the performance of macrophages and their ability to phagocytose newly implanted endometriotic lesions [37]. It is then suggested that the microbiota, particularly in a state of dysbiosis, may contribute to immune activation that favors peritoneal inflammation and possibly the progression of endometriosis. The study by Wang et al. [29], included in this review, evaluated the presence of inflammatory markers in the peritoneal fluid. After the analysis, it was detected that the levels of IL-6, IL-10, IL-13, and TNF-*α* were significantly higher in women with endometriosis and infertility (*P* < 0.05).

In order to survive, endometriotic implants need a blood supply, and this angiogenesis process is regulated by factors such as vascular endothelial growth factor. In peritoneal fluid, vascular endothelial growth factor is produced mainly by macrophages, and its expression is directly regulated by estradiol and progesterone. TNF-*α* and IL-8, also secreted by peritoneal macrophages, are other potent inducers of angiogenesis and lesion proliferation. The role of TNF-*α* in stimulating endometrial cell adhesion and inducing angiogenesis is particularly needed in the early stages of the onset of endometriosis [38, 39].

One of the theories about the origin of ectopic endometrial tissues is the “retrograde menstruation theory”, in which the reflux of menstrual debris with viable endometrial cells into the pelvic cavity occurs, resulting in adhesion to peritoneal surfaces and proliferation [40]. Garcia-Valesco and Arici [41] have shown that in an inflammatory peritoneal environment, this adhesion is more intensified. Khan et al. [42] propose the hypothesis of bacterial contamination. The authors suggest that a substantial amount of endotoxin in the peritoneal fluid, related to menstrual blood reflux, would be involved in pelvic inflammation and promote Toll-like receptor 4- (TLR4-) mediated growth and progression of endometriosis.

Thus, lipopolysaccharide (LPS), a component of the outer membrane of gram-negative bacteria, can be the initial trigger and bacterial contamination its source in the intrauterine environment, and it could be the primary cause in the regulation of endometriosis growth, alone or in combination with ovarian steroids [42]. Most studies included in this systematic review showed an increase in gram-negative bacteria in women with endometriosis in either intestinal, cervical, or vaginal samples [19, 21–23, 25, 26, 29, 33], corroborating Khan et al.'s [42] hypothesis.

Another hypothesis in which the intestinal microbiota could be related to endometriosis is through the regulation of estrogen. This process would be linked to estrobolome, defined by Plottel and Blaser [43] as “the aggregate of enteric bacterial genes whose products are capable of metabolizing estrogens.” Thus, intestinal microbial richness would regulate systemic estrogen levels through the action of *β*-glucuronidase, present in some species of bacteria. In the intestine, this enzyme converts hepatically conjugated estrogens, which would otherwise be excreted to their active form, leading to their reabsorption [44].

Therefore, in a state of dysbiosis, there would be an increase in systemic levels of estrogen, which can be transported to distal mucosal sites, such as the endometrium [44]. One of the bacteria that presents *ß*-glucuronidase activity is the *Escherichia coli* species, a bacterium belonging to the phylum *Proteobacteria*. In our review, the studies by Nabiel et al. [26], Khan et al. [23], and Akiyama et al. [21] showed a significant increase in *E. coli* species in women with endometriosis. Besides, the study of Chang et al. [33] indicated the presence of *Proteobacteria* members as one unique population in the cervix of women with endometriosis.

A dysregulation of the balance between the ratio of bacteria belonging to the *Firmicutes* and *Bacteroidetes* phyla is presented in women with endometriosis and may be acting in the deregulation of estrogen metabolism since the bacteria in these phyla have genes related to glucuronidase [45, 46]. The increase in bacteria belonging to the phylum *Firmicutes* was presented in studies by Khan et al. [23], Hernandes et al. [22], Lee et al. [25], Shan et al. [27], and Chang et al. [33], that last one also indicating the increase in *Firmicutes* community in infertile patients as compared to fertile patients, especially *Lactobacillus.*

Analyses of studies included in this systematic review indicate that another aspect to be considered is the relationship between microbiota and the stage of endometriosis. As evidenced by Ata et al. [19], highlighting the complete absence of Atopobium (belonging to the phylum *Actinobacteria*) in the vaginal and cervical microbiota of the endometriosis group in stages 3 and 4. Furthermore, in the cervical microbiota, *Gardnerella*, *Streptococcus*, *Escherichia*, *Shigella*, and *Ureoplasma* were increased in stages 3 and 4, all containing potentially pathogenic species.

The results of the study by Perrotta et al. [20] also suggested that the vaginal microbiota can predict disease stage since an OTU within the *Anerococcus* genus differed significantly in abundance between women in stages 1 and 2 and stages 3 and 4 of endometriosis during the menstrual phase.

Cregger et al. [47] identified 56 significantly different OTUs on the day of surgery between the stage 3 patient with endometriosis and all others analyzed. Suggesting that changes in the bacterial community level may be indicative of severe active endometriosis and provide an additional explanation for the decrease in pregnancy rates in these women, suggesting that the analysis of the bacterial community profile could help in the diagnosis of endometriosis in asymptomatic infertile women.

Moreno et al. [48] suggested that non-*Lactobacillus*-dominant compositions may trigger an inflammatory response in the endometrium, which may be an explanation for their association with negative pregnancy outcomes. Nine studies included in this systematic review [19, 21–24, 26, 31–34] evaluated *Lactobacillus* spp. in cervical, endometrial, or vaginal samples; however, due to differences in the presentation of results, it was not possible to determine whether a difference occurred between women with endometriosis and women in control groups.

Leonardi et al. [7] also performed a systematic review of the endometriosis-microbiome interaction in human and animal studies. The results found in our systematic review and meta-analysis corroborate the results found in the systematic review by Leonardi et al. [7], in which the authors stated that endometriosis seems to be associated with an increased presence of *Proteobacteria*, *Enterobacteriaceae*, *Streptococcus* spp., and *Escherichia coli*, and also they indicated an association between the increase in the phylum *Firmicutes* and the genus *Gardnerella*. However, our review differs from theirs as our assessment has included studies only with humans, including intestinal, vaginal, cervical, endometrial, and peritoneal fluid microbiota.

Recently, a literature review published by Talwar et al. [49] carried out a synthesis of the processes involved in the pathogenesis of endometriosis and its relationship with the intestinal microbiota, indicating that a reduced gut microbiome diversity and an elevated *Firmicutes/Bacteroidetes* ratio have mostly been linked with increased endometriosis risk, contributing to highlight our results. Similarly to our study, Talwar et al. [49] demonstrated the results of studies evaluated in our systematic review [19, 20, 27, 28, 32], presenting their strengths and limitations. In addition to that, our systematic review contributes by bringing the quality assessment of these studies and the quantitative analysis of the results through meta-analysis.

Most of the studies included in this review analyzed the intestinal microbiota, which was to be expected, since studies on microbiota started by evaluating the gastrointestinal tract. In recent years, other organs and fluids have also begun to have their microbiota evaluated. In our study, the cervical, vaginal, endometrial, and peritoneal fluid microbiota were also analyzed. These new analyses become important for clinical practice, enabling a greater understanding of the disease and the search for new forms of treatment for women with endometriosis.

The analysis technique used in the included studies was the 16S rRNA genomic extraction methodology. However, in recent years, there has been an improvement in culture methods, defined as “culturomics”, consisting of the use of different culture conditions, such as the use of selective liquid and/or solid media for microorganism growth, variable temperature, and time of incubation [50, 51]. Metagenomics and culturomics are different analytical methods that pursue the same objective: the detection of as many microbial species in a particular ecosystem as possible. As a strong point, the possibility of analyzing a high number of samples influences the speed of analysis results [51]. On the other hand, it is still a technique that presents limitations, such as the efficacy of DNA extraction is a crucial operator-dependent step that could affect the repeatability of the test [50]. This shows that although we are making progress in microbiota research, we still have limitations to overcome.

### 4.3. Strengths and Limitations

The results presented here indicate that in women with endometriosis, there are changes in relation to the species/genera of bacteria in the microbiota. These findings show the importance of including the analysis and management of the microbiota in clinical practice, as well as the importance of studies in this area.

As limitations to this study, it is highlighted that the microbiota is directly influenced by lifestyle, socioeconomic, cultural, environmental, and genetic factors. Therefore, primary studies should search for ways to minimize these interferences or find ways to incorporate them as a measurable factor. In addition to these factors, there are also limitations in the methodologies of the studies, with no standardization regarding which phase of the menstrual cycle the samples are collected, hormonal levels, and the presentation of the results of the analyzed species, resulting in substantial heterogeneity.

## 5. Conclusions

Alpha diversity was not significantly different for intestinal and vaginal microbiota in women with endometriosis compared to women without endometriosis. However, when analyzing the genus/species in the composition of the microbiota in women with endometriosis, we found a possible relationship that must be further investigated.

## Figures and Tables

**Table 1:** Characteristics of included studies.

Author/year	Country	Study design	*N* control		*N* endometriosis		Diagnostic criteria for endometriosis	Sample evaluated (intestinal, vaginal, cervical) with quantity (*n*)	
			*n*	Age	N	Age		Control	Endometriosis
Akiyama et al., 2019 [21]	Japan	Case-control	39	Mean ± SD: 32.5 ± 6.0	30	Mean ± SD: 33.9 ± 5.7	Diagnosed by laparoscopy and confirmed by pathology; the stages were classified according to the revised American Society for Reproductive Medicine (r-ASRM) scoring system	Cervical (*n* = 39)	Cervical (*n* = 30)
Ata et al., 2019 [19]	Turkey	Cohort	14	Median (IQR): 27.5 (25.8-30)	14	Median (IQR): 28.5 (26-31.3)	Histological diagnosis	Intestinal, cervical, and vaginal (*n* = 14)	Intestinal, cervical, and vaginal (*n* = 14)
Chang et al., 2022 [33]	Taiwan	Case-control	10	NA	23	24–54	Diagnosed by laparotomy or laparoscopy and pathologically proven as endometriosis	Cervical (*n* = 10)	Cervical (*n* = 23)
Hernandes et al., 2020 [22]	Brazil	Case-control	11	NA	10	NA	Lesion(s) identification by laparoscopic surgery, and further confirmation by histopathology analysis	Endometrial (*n* = 18); endometriotic Lesion (*n* = 8); vaginal fluid (*n* = 21)	
Huang et al., 2021 [32]	China	Case-control	20	Mean ± SD: 34.0 (±10.8)	21	Mean ± SD: 38.3 (±7.88)	Confirmation by laparoscopy with biopsy analysis	Feces (*n* = 40); cervical mucus (*n* = 38); peritoneal fluid (*n* = 41)	
Khan et al., 2014 [23]	Japan	Case-control	55	Mean ± SD: 35.8 ± 7.9	73	Mean ± SD: 36.5 ± 7.1	Elective laparoscopy for infertility or diagnostic laparoscopy for dysmenorrhea and subsequently confirmed by histology	Endometrial (*n* = 53); vaginal (*n* = 6)	Endometrial (*n* = 65); vaginal (*n* = 10)
Khan et al., 2016 [24]	Japan	Case-control	32 (16 under treatment with GnRHa/16 without treatment with GnRHa)	No treatment (mean ± SD): 33.6 ± 8.9 Treatment group (mean ± SD): 42.1 ± 8.6	32 (16 under treatment with GNRHa/16 without treatment with GnRHa)	No treatment (mean ± SD): 35.7 ± 8.3 Treatment group (mean ± SD): 37.5 ± 5.6	Elective laparoscopy for infertility or diagnostic laparoscopy for dysmenorrhea and subsequently confirmed by histology	Endometrial (*n* = 32)	Endometrial (*n* = 32); cystic fluid from ovarian endometrioma (*n* = 8); nonendometrioma cystic fluid (*n* = 8)
Khan et al., 2021 [31]	Japan	Case-control	11 (no treatment group)	(Mean ± SD) 41.2 ± 8.1 (no treatment group)	21 (no treatment group)	(Mean ± SD) 36.3 ± 7.7 (no treatment group)	Elective laparoscopy for infertility or diagnostic laparoscopy for dysmenorrhea and subsequently confirmed by histology	Entrometrial and endometriotic samples (*n* = 11)	Endometrial and endometriotic samples (*n* = 21)
Lee et al., 2021 [25]	South Korea	Case-control	45	Mean (standard error): 394 ± 1.1	45	Mean (standard error): 36.2 ± 1.3	Confirmation by histological evaluation; the stages were classified according to the revised American Society for Reproductive Medicine (r-ASRM) scoring system	Peritoneal fluid (*n* = 45)	Peritoneal fluid (*n* = 45)
Nabiel et al., 2020 [26]	Egypt	Case-control	51	Median (IQR): 36 (25–48)	51	Median (IQR): 35 (22–49)	Laparoscopy followed by histopathological evaluation for diagnosis confirmation; the stages and severity were classified according to the revised American Society for Reproductive Medicine (r-ASRM) scoring system	Endometrial (*n* = 51)	Endometrial (*n* = 51)
Perrotta et al., 2020 [20]	Brazil/USA	Cross-sectional	24	Mean ± SD: 35.25 (6.9)	35	Mean ± SD: 34.9 (6.8)	Diagnosis by laparoscopy and imaging (transvaginal ultrasound or magnetic resonance)	Rectal (*n* = 24); vaginal (*n* = 24)	Rectal (*n* = 35); vaginal (*n* = 35)
Shan et al., 2021 [27]	China	Case-control	12	(32 ± 2 vs. 32 ± 3 years, *P* > 0.05) Age of each group unspecified	12	(32 ± 2 vs. 32 ± 3 years, *P* > 0.05) Age of each group unspecified	Confirmation by histological evaluation; the stages and severity were classified according to the revised American Society for Reproductive Medicine (r-ASRM) scoring system	Intestinal (*n* = 12)	Intestinal (*n* = 12)
Svensson et al., 2021 [28]	Sweden	Case-control within a cohort study	198	Median (IQR): 37.0 (32.0–44.0)	66	Median (IQR): 37.8 (32.8–43.3)	ICD-10 classification for endometriosis (N80); diagnosis confirmed by laparoscopy or laparotomy	Intestinal (*n* = 198)	Intestinal (*n* = 66)
Wang et al., 2018 [29]	China	Case-control	30	Mean ± SD: 37.7 ± 7.4	55	Mean ± SD: 37.2 ± 8.2	Diagnosis by laparoscopy	Peritoneal fluid (*n* = 30)	Peritoneal fluid (*n* = 55)
Wei et al., 2020 [30]	China	Cohort	14	NA	36	NA	Endometriosis determined according to American Society for Reproductive Medicine (r-ASRM) criteria	Vaginal (*n* = 14); cervical mucus (*n* = 14); endometrial (*n* = 11); peritoneal fluid (*n* = 14)	Vaginal (*n* = 36); cervical mucus (*n* = 36); endometrial (*n* = 26); peritoneal fluid (*n* = 36)
Yuan et al., 2022 [34]	China	Case-control	25	33.32 ± 8.04	36	35.28 ± 7.24	Diagnosis based on laparoscopy and pathology confirmation	Peritoneal fluid (*n* = 25)	Peritoneal fluid (*n* = 36)

Legend: SD: standardized deviation; IQR: interquartile range; NA: not available; ICD: International Classification of Diseases; GnRHa: gonadotropin-releasing hormone agonist; LVFX: levofloxacin.

**Table 2:** Alpha diversity analysis of included studies.

Author/year	Alpha diversity analysis (*mean* ± *SD*)
Akiyama et al., 2019 [21]	**Shannon index**-*cervical sample* Control group: 3.81 ± 1.11; endometriosis group: 5.39 ± 1.27

Ata et al., 2019 [19]	**Shannon index** *Vaginal sample*-control group: 0.5 ± 0.36; endometriosis group: 0.43 ± 0.32 *Cervical sample*-control group: 0.59 ± 0.46; endometriosis group: 0.46 ± 0.31 *Intestinal sample*-control group: 3.75 ± 0.23; endometriosis group: 3.81 ± 0.26

Chang et al., 2022 [33]	**Shannon index** Assessed against the CA125 marker for infertility and endometriosis symptoms.

Hernandes et al., 2020 [22]	**Shannon index**-*vaginal fluid* Control group: 0.88 ± 0.40 Endometriosis group: 0.38 ± 0.36
	**Shannon index**-*endometrium* Control group: 1.83 ± 0.67 Endometriosis group: 0.33 ± 0.34
	**Simpson index-** *vaginal fluid* Control group: 0.69 ± 0.25 Endometriosis group: 1.10 ± 0.27
	**Simpson index**-*endometrium* Control group: 0.42 ± 0.28 Endometriosis group: 1.19 ± 0.23

Huang et al., 2021 [32]	**Shannon index**-*fecal sample* Control group: 2.66 ± 0.21 Endometriosis group: 2.38 ± 0.35
	**Simpson index**-*fecal sample* Control group: 0.88 ± 0.03 Endometriosis group: 0.83 ± 0.07
	**Shannon index**-*cervical mucus* Control group: 1.13 ± 0.92 Endometriosis group: 0.86 ± 0.58
	**Simpson index**-*cervical mucus* Control group: 0.44 ± 0.29 Endometriosis group: 0.39 ± 0.27
	**Shannon index**-*peritoneal fluid* Control group: 3.78 ± 1.58 Endometriosis group: 3.71 ± 1.43
	**Simpson index**-*peritoneal fluid* Control group: 0.84 ± 0.31 Endometriosis group: 0.85 ± 0.25

Khan et al., 2014 [23]	Not evaluated in the study.

Khan et al., 2016 [24]	Not evaluated in the study.

Khan et al., 2021 [31]	**Shannon index**-*endometrium-*median (CI)^∗^ Control group (untreated): 2.59 (1.91-2.95) Endometriosis group (untreated): 3.02 (2.63-3.44)

Lee et al., 2021 [25]	**Shannon index-** *peritoneal fluid* Control group: 4.35 ± 0.31 Endometriosis group: 4.52 ± 0.23
	**Simpson index-** *peritoneal fluid* Control group: 0.66 ± 0.21 Endometriosis group: 0.77 ± 0.20

Nabiel et al., 2020 [26]	Not evaluated in the study.

Perrotta et al., 2020 [20]	Not evaluated in the study.

Shan et al., 2021 [27]	**Shannon index-** *intestinal sample* Control group: 2.97 ± 0.24 Endometriosis group: 2.93 ± 0.21
	**Simpson index-** *intestinal sample* Control group: 0.092 ± 0.205 Endometriosis group: 0.084 ± 0.204

Svensson et al., 2021 [28]	**Shannon index-** *intestinal sample* Control group: 3.34 ± 0.20 Endometriosis group: 3.30 ± 0.20

Wang et al., 2018 [29]	Not evaluated in the study.

Wei et al., 2020 [30]	Not evaluated in the study.

Yuan et al., 2022 [34]	**Shannon index**-*peritoneal fluid-*median (IC)^∗^ Control group: 1.65 (1.59-1.68) Endometriosis group: 1.55 (1.46-1.63)
	**Simpson index**-*peritoneal fluid -* median (IC)^∗^ Control group: 0.32 (0.31-0.33) Endometriosis group: 0.32 (0.32-0.33)

SD: standard deviation. ^∗^Data extracted from the graphs presented in the study.

**Table 3:** Methods and results of included studies.

Author/year	Microbiota analysis method	Taxonomy of the microbiota present in women with endometriosis (genus/family)	Comparison of the microbiota present in women with endometriosis and the control group (%)	Assessment of microbiota diversity in women with endometriosis compared to control group	Main results
Akiyama et al., 2019 [21]	DNA extraction with 16S rRNA gene amplification and sequencing of V5-V6 regions	*Corynebacterium* (genus); *Enterobacteriaceae* (family); *Flavobacterium* (genus); *Pseudomonas* (genus); *Streptococcus* (genus); *Lactobacillus* (genus); *Prevotella* (genus); *Gardnerella* (genus)	*Corynebacterium* Control: 0.15%; endometriosis: 1.38% *Enterobacteriaceae* Control: 0.90%; endometriosis: 2.22% *Flavobacterium* Control: 3.30%; endometriosis: 5.38% *Pseudomonas* Control: 1.53%; endometriosis: 3.26% *Streptococcus* Control: 0.1%; E endometriosis: 3.75% *Lactobacillus* Control: 50.12%; endometriosis: 39.51% *Prevotella* Control: 9.88%; endometriosis: 6.95%	**Alpha diversity-Shannon index ( ** **m** **e** **a** **n** ± **SD)** Control: 3.81 ± 1.11; endometriosis: 5.39 ± 1.27	In addition to the predominance of *lactobacilli* spp., populations of *Corynebacterium*, *Enterobacteriaceae*, *Flavobacterium*, *Pseudomonas*, and *Streptococcus* were increased in the endometriosis group. The genera *Enterobacteriaceae* and *Streptococcus* were identified as more significant candidates in the endometriosis group than in controls (*P* < 0.05 for each).

Ata et al., 2019 [19]	DNA extraction with 16S rRNA gene amplification and sequencing of V3-V4 regions	**Fecal sample** *Barnesiella* (genus); *Sneathia* (genus) **Cervical sample** *Alloprevotella* (genus) **Vaginal sample** *Gemella* (genus) **Vaginal microbiota** *Gardnerella* (genus); *Lactobacillus* (genus) **Cervical microbiota** *Gardnerella* (genus); *Lactobacillus* (genus); *Prevotella* (genus) **Gut microbiota** *Bacteroides* (genus); *Faecalibacterium* (genus); *Escherichia/Shigella* (genus); *Prevotella* (genus); *Roseburia* (genus); *Blautia* (genus); *Lachnospiracea* (genus); *Dialister* (genus); *Ruminococcus* (genus); *Bifidobacteium* (genus); *Alistipes* (genus)	**Abundance rate (%)** **Vaginal microbiota** *Gardnerella* Control: 7.23%; Endometriosis: 10.47% *Gardnerella* (significant after the exclusion of *Lactobacillus*)– Control: 36.8% Endometriosis: 72.9% (*P* < 0.05) **Cervical microbiota** *Gardnerella* Control: 7.24%; Endometriosis: 10.18% *Gardnerella* (significant after the exclusion of *Lactobacillus*) Control: 36.8% Endometriosis: 67.7% (*P* < 0.05) *Prevotella* Control: 5.09%; endometriosis: 3.75%	**Alpha diversity-Shannon index ( ** **m** **e** **a** **n** ± **SD)** **Vaginal sample** Control: 0.5 ± 0.36; endometriosis: 0.43 ± 0.32 **Cervical sample** Control: 0.59 ± 0.46; endometriosis: 0.46 ± 0.31 **Intestinal sample** *Escherichia*/*Shigella* (gênero) Control: 3.75 ± 0.23; endometriosis: 3.81 ± 0.26	Although the general composition of the vaginal, cervical, and intestinal microbiota was similar between the stage 3/4 endometriosis group and the control group, differences in genus levels were observed. The complete absence of *Atopobium* in the vaginal and cervical microbiota of the stage 3/4 endometriosis group was noted. In the cervical microbiota, *Gardnerella, Streptococcus*, *Escherichia*, *Shigella*, *and Ureoplasma*, all containing potentially pathogenic species, were increased in stage 3/4 endometriosis. In addition, more women in the stage 3/4 endometriosis group had *Shigella/Escherichia* dominant gut microbiome

Chang et al., 2022 [33]	DNA extraction with amplification by PCR of the regions V3, V4, V5, and V9) of the 16S ribosomal RNA (rRNA)	*Firmicutes* (phylum) *Actinobacteria* (phylum) *Bacteroidetes* (phylum) *Proteobacteria* (phylum) *Fusobacteria* (phylum) *Tenericutes* (phylum) *Spirochaetes* (phylum) *Chlamydiae* (phylum) *Synergistetes* (phylum) *Cyanobacteria* (phylum)	**Relative abundance** **Endometriosis group–Stage I-II** Firmicutes: 0.649 Actinobacteria: 0.1826 Bacteroidetes: 0.063 **Endometriosis group–Stage III-IV** Firmicutes: 0.773 Actinobacteria: 0.086 Bacteroidetes: 0.052 **Control group:** Firmicutes: 0.611 Actinobacteria: 0.205 Bacteroidetes: 0.095	Shannon index evaluated for CA125 marker associated with female fertility and pain score	Cervical microbiome can be altered during endometriosis development and progression with a tendency of increased *Firmicutes* and decreased *Actinobacteria* and *Bacteroidetes*. Distinct from vaginal microbiome, upregulation of *Lactobacillus*, in combination with increased *Streptococcus* and decreased *Dialister,* was frequently associated with advanced endometriosis stages, DIE, higher CA125 levels, severe pain, and infertility. Significantly, reduced richness and diversity of the cervical microbiome were detected in patients with more severe clinical symptoms.

Hernandes et al., 2020 [22]	DNA extraction with 16S rRNA gene amplification and sequencing of V3-V4 regions	**Vaginal fluid** *Lactobacillus* (genus); *Gardnerella* (genus); *Streptococcus* (genus); *Prevotella* **Endometrium** *Lactobacillus* (genus); **Endometriotic lesion** *Lactobacillus* (genus); *Enterococcus* (genus); *Gardnerella* (genus); *Pseudomonas* (genus); *Alishewanella* (genus); *Ureaplasma* (genus); *Aerococcus* (genus)	**Abundance rate (%)** **Vaginal fluid** *Lactobacillus* Control: 75.85 ± 16.72%; endometriosis: 90.77 ± 9.64% **Endometrium** *Lactobacillus* Control: 36.36 ± 18.37%; endometriosis: 92.75 ± 6.70%	**Alpha diversity-Shannon index** **( ** **m** **e** **a** **n** ± **SD)** **Shannon-vaginal fluid** Control: 0.88 ± 0.40 Endometriosis: 0.38 ± 0.36 **Shannon-Endometrum** Control: 1.83 ± 0.67 Endometriosis: 0.33 ± 0.34	The sequencing analysis resulted in similar profiles of microorganisms, composed more abundantly by the genera *Lactobacillus*, *Gardnerella*, *Streptococcus,* and *Prevotella*. No significant difference was found in the analysis of the diversity of microbiome profiles between control and endometriotic patients; however, deep endometriotic lesions seem to have a different bacterial composition, with less predominant *Lactobacillus* and with *Alishewanella*, *Enterococcus*, and *Pseudomonas* more abundant.

Huang et al., 2021 [32]	DNA extraction with amplification and sequencing of V4 regions of the 16S rRNA gene	*Firmicutes* (phylum) *Bacteroidetes* (phylum) *Proteobacteria* (phylum) *Actinobacteria* (phylum) *Acidobacteria* (phylum)	**Cervical mucus** *Lactobacillus* Control: 0.34 ± 0.38 Endometriosis: 0.37 ± 0.45	**Beta diversity** **Fecal sample–Shannon index** Control: 2.66 ± 0.21 Endometriosis: 2.38 ± 0.35 **Simpson index** Control: 0.88 ± 0.03 Endometriosis: 0.83 ± 0.07 **Cervical mucus–Shannon index** Control: 1.13 ± 0.92 Endometriosis: 0.86 ± 0.58 **Simpson index** Control: 0.44 ± 0.29 Endometriosis: 0.39 ± 0.27 **Peritoneal fluid–Shannon index** Control: 3.78 ± 1.58 Endometriosis: 3.71 ± 1.43 **Simpson index** Control: 0.84 ± 0.31 Endometriosis: 0.85 ± 0.25	Endometriosis patients harbor distinct microbial communities versus the control group, especially in feces and peritoneal fluid, with an increased abundance of pathogens in peritoneal fluid and a depletion of protective microbes in feces. *Ruminococcus* and *Pseudomonas* genera were identified as potential biomarkers in gut and peritoneal fluid, respectively.

Khan et al., 2014 [23]	Endometrial sample: culture with LBS agar selective for aerobic limbs *Lactobacillus*; brain heart infusion agar (BHI) for aerobic and facultative anaerobic gram (+); eosin-methylene agar or Luria-Bertani broth for gram (-); sabouraud chloramphenicol agar for isolating fermenters; incubation for 48 h 5% CO_2_	**Vaginal sample-prevalence of positive endometrial cultures and acute endometritis according to the presence or absence of altered vaginal flora** *Mobiluncus* (genus); *Gardnerella* (genus); *Lactobacillus* (genus); *Enterococcus* (genus); *Escherichia* (genus); *Staphylococcus* (genus); *Streptococcus* (genus)	**Prevalence of positive endometrial cultures and acute endometritis according to the presence or absence of altered vaginal flora** *Mobiluncus* score 1–2 Endometriosis: presence: 70.1%; absence: 25.9% Endometritis: presence: 65.2%; absence: 34.7% (*P* 0.12) *Gardnerella* score 3–4 Endometriosis: presence: 77.8%; absence: 22.3% Endometritis: presence: 78.3%; absence: 21.7% (*P* 0.02) *Lactobacillus* score 3–4 Endometriosis: presence: 59.3%; absence: 40.7% Endometritis: presence: 60.8%; absence: 39.2% (*P* 0.28)	NA	Nine different types of microbial species were discovered; colony formations of *Gardnerella*, *α-Streptococcus*, *Enterococci*, and *E. coli* were significantly higher in endometrial samples derived from women with endometriosis than in control women (*P* < 0.05 for each species). Endometrial samples derived from women with endometriosis were more likely to have bacterial colonization (*P* = 0.03) than from control women.

Khan et al., 2016 [24]	DNA extraction with amplification and sequencing of the 16S rRNA gene (16S rDNA) in species-specific regions	*Lactobacillaceae* (famíly); *Streptococcaceae* (famíly); *Staphylococcaceae* (famíly); *Enterobacteriaceae* (famíly); *Moraxellaceae* (famíly)	Endometrial sample *Lactobacillaceae* Control: 24.04%; endometriosis: 18.79% *Streptococcaceae* Control: 5.48%; endometriosis: 15.52% *Staphylococcaceae* Control: 16.46%; endometriosis: 10.85% *Enterobacteriaceae* Control: 12.37%; endometriosis: 3.27% *Moraxellaceae* Control: 2.68%; endometriosis: 8.64% Endometrioma/nonendometrioma cystic fluid sample (endometriosis group only) *Lactobacillaceae* Endometrioma-3.06% Nonendometrioma-7.50% (*P* < 0.05) *Streptococcaceae* Endometrioma-9.19% Nonendometrioma-0.40% (*P* < 0.01) *Staphylococcaceae* Endometrioma-6.53% Nonendometrioma-2.09% (*P* < 0.05) *Enterobacteriaceae* Endometrioma-0.48% Nonendometrioma-1.85% *Moraxellaceae* Endometrioma-13.25% Nonendometrioma -10.32% (*P* < 0.074)	NA	The 16S metagenoma assay detected a significantly higher percentage of *Streptococcaceae* (*P* < 0.01) and *Staphylococcaceae* (*P* < 0.05) in cystic fluid derived from women with ovarian endometrioma compared to that in cystic fluid collected from nonendometrial cysts

Khan et al., 2021 [31]	DNA extraction with broad-range polymerase chain reaction (PCR) amplification of bacteria targeting the V5-V6 region of the 16S rRNA gene.	*Firmicutes* (phylum) *Acidobacteriota* (phylum) *Pseudomonadota* (phylum) *Actinobacteria* (phylum) *Bacteroidota* (phylum) *Actinomycetota* (phylum) *Bacillota* (phylum) *Fusobacteriota* (phylum)	**Endometrial samples-untreated endometriosis group and untreated control group** *Lactobacillus* Endometriosis: 21.82%; control: 35.92% *Gardnerella* Endometriosis: 11.90%; control: 13.75% *Prevotella* Endometriosis: 13.61%; control: 8.91% *Sneathia* Endometriosis: 1.77%; control: 2.25% *Acidibactor* Endometriosis: 11.71%; control: 7.05% *Atopobium* Endometriosis: 3.47%; control: 2.48% *Megasphoera* Endometriosis: 3.91%; control: 3.07% *Streptococcus* Endometriosis: 4.78%; control: 3.86% *Bradyrhizobium* Endometriosis: 8.58%; Control: 3.09%	**Alpha diversity-untreated endometriosis group and untreated control group** **Shannon index** Endometriosis group–median (CI) 3.02 (2.63–3.44) Control group–median (CI) 2.59 (1.91–2.95)	The beta diversity results indicate that the microbiota in endometrial samples could depend on the individuals regardless of treatment. In the nontreatment group, the alpha diversity in the endometriosis group was significantly higher (*P* = 0.036) than in the control group. In women with endometriosis, treatment with either of LVFX or GnRHa + LVFX significantly decreased *Gardnerella*, *Prevotella*, *Acidibactor*, *Atopobium*, *Megasphaera*, and *Bradyrhizobium* (*P* < 0.05 for each) comparing to untreated group.

Lee et al., 2021 [25]	DNA extraction with amplification and sequencing of the V3-V4 regions of the 16S rDNA gene	*Actinobacteria* (phylum); *Acinetobacter* (genus); *Pseudomonas* (genus); *Propionibacterium* (genus); *Streptococcus* (genus); *Rothia (genus);* *Actinomyces* (genus); *Enhydrobacter* (genus); *Firmicutes* (phylum); *Proteobactecia* (phylum); *Verrucomicrobia* (phylum); *Bacteroidetes* (phylum); *Deferribacteres* (phylum); *Fusobacteria* (phylum); *Cyanobacteria* (phylum); *Tenericutes* (phylum); *Armatimonadetes* (phylum); *Thermi* (phylum); *Euryarchaeota* (phylum); *Chloroflexi* (phylum); *Spirochaetes* (phylum); *Planctomycetes* (phylum); *Acidobacteria* (phylum); *Gemmatimonadetes* (phylum); *Synergistete* (phylum)s; *Lentisphaerae (phylum)*	**Composição microbiana por taxonomia ( ** **m** **e** **d** **i** **a** ± **DP) gênero** *Acinetobacter* Endometriosis: 0.0602 ± 0.0265 Control: 0.0416 ± 0.0289(*P* 0.0022) *Pseudomonas* Endometriosis: 0.0325 ± 0.0235 Control: 0.0208 ± 0.0175 (*P* 0.0097) *Propionibacterium* Endometriosis: 0.0049 ± 0.0062 Control: 0.0129 ± 0.0190 (*P* 0.0111) *Streptococcus* Endometriosis: 0.0291 ± 0.0231 Control: 0.0179 ± 0.0204 (*P* 0.0183) *Rothia* Endometriosis: 0.0018 ± 0.0045 Control: 0.0115 ± 0.0261 (*P* 0.0190) *Actinomyces* Endometriosis: 0.0017 ± 0.0043 Control: 0.0126 ± 0.0305 (*P* 0.0233) *Enhydrobacter* Endometriosis: 0.0071 ± 0.0114 Control: 0.0026 ± 0.0072 (*P* 0.0300)	**Alpha diversity-indexes ( ** **m** **e** **a** **n** ± **SD)** Observed Control: 196.90 ± 39.00; endometriosis: 208.44 ± 22.08 Chao1 Control: 348.12 ± 41.13; endometriosis: 341.25 ± 34.64 Shannon Control: 4.35 ± 0.31; endometriosis: 4.52 ± 0.23 Simpson Control: 0.66 ± 0.21; endometriosis: 0.77 ± 0.20	At the genus level, the abundance of *Acinetobacter*, *Pseudomonas*, *Streptococcus*, and *Enhydrobacter* increased significantly, while the abundance of *Propionibacterium*, *Actinomyces*, and *Rothia* decreased significantly in the endometriosis group compared to the control group (*P* < 0.05).

Nabiel et al., 2020 [26]	Culture with Columbia agar 5% sheep blood, chocolate agar, mannitol salt agar, MacConkey agar, brain-heart infusion agar, and LBS agar; RNA extraction with complementary DNA transcription	*Lactobacilli* (genus); *G. vaginalis* (species); *α-Streptococcus* (genus); *S. agalactiae* (species); *S. aureus* (species); *Enterococci* (genus); *Mobiluncus* (genus); *E. coli.* (species)	**Abundance rate (%)** *Lactobacilli* Endometriosis: 74.5% Control: 68.6% *G. vaginalis* Endometriosis: 45.1% Control: 7.8% (*P* < 0.0001) *α-Streptococcus* Endometriosis: 64.7% Control: 66.7% *S. agalactiae* Endometriosis: 41.2% Control: 17.7% (*P* 0.009) *S. aureus* Endometriosis: 53.2% Control: 23.5% (*P* 0.001) Enterococci- Endometriosis: 66.7% Control: 54.9% *Mobiluncus* Endometriosis: 39.2% Control: 7.8% (*P* < 0.0001) *E. coli.* Endometriosis: 70.6% Control: 41.2% (*P* 0.005)	NA	The presence of *G. vaginalis*, *S. agalactiae*, *S. aureus*, *Mobiluncus*, and *E. coli* in the endometrium was significantly associated with endometriosis (*P* < 0.0001, 0.009, 0.001, <0.0001, and 0.005, respectively), while *Lactobacilli*, *α-Streptococcus,* and *Enterococcus* were not associated.

Perrotta et al., 2020 [20]	DNA extraction with sequencing of the V4 region of the 16S rRNA gene	*Anaerococcus* (genus); *L. crispatus* (species)	NA	NA	The distribution of vaginal community state status (CSTs) differed significantly between the follicular and menstrual phases of the menstrual cycle (*P* = 0.021).

Shan et al., 2021 [27]	DNA extraction with amplification and sequencing of the 16sRNA V3-V4 regions	*Bacteroides* (genus); *Faecalibacterium* (genus); *Bifidobacterium* (genus); *Blautia* (genus); *Roseburia* (genus); *Prevotella* (genus); *Subdoligranulum* (genus); *Lachnospira* (genus); *Fusicatenibacter* (genus); *Escherichia-Shigella* (genus); *Dorea* (genus); *Streptococcus* (genus); *Eubacterium eligens* (genus); *Lachnoclostridium* (genus); *Phascolarctobacterium* (genus); *Peptostreptococcaceae* (genus); *Anaerostipes* (genus); *Ruminococcus* (genus); *Klebsiella* (genus); *Eubacterium hallii* (genus)	*Bifidobacterium* Control: 3.49%; endometriosis: 14.65% (*P* = 0.01529) *Blautia* Control: 3.02%; endometriosis: 13.02% (*P* = 0.000308) *Lachnospira* Control: 5.11%; endometriosis: 0.23% (*P* = 0.00007631) *Dorea* Control: 0.70%; endometriosis: 3.95% (*P* = 0.008601) *Streptococcus* Control: 0.70%; endometriosis: 3.95% (*P* = 0.0179) [*Eubacterium]_eligens_group* Control: 3.95%; endometriosis: 0.23% (*P* = 0.0004872) *Eubacterium_hallii _group* Control: 0.70%; endometriosis: 2.55% (*P* = 0.02258)	**Alpha diversity-indexes ( ** **m** **e** **a** **n** ± **SD)** Sobs Control: 176.00 ± 18.04; endometriosis: 171.34 ± 9.58 Ace Control: 196.82 ± 19.24; endometriosis: 207.93 ± 14.65 Shannon Control: 2.97 ± 0.24; endometriosis: 2.93 ± 0.21 Simpson– Control: 0.092 ± 0.205; endometriosis: 0.084 ± 0.204	No significant difference was found in the *α* diversity analysis (*P* > 0.05). In the abundance analysis, 20 main genera were identified. The genera *Bifidobacterium*, *Blautia*, *Dorea*, *Streptococcus*, and *[Eubacterium] hallii_group* showed a significantly higher abundance in the endometriosis group compared to the control group, while *Lachnospira* and *[Eubacterium]_eligens_group* showed a significantly lower abundance in the endometriosis group

Svensson et al., 2021 [28]	DNA extraction with 16S rRNA sequencing in V1-V3 regions	*Paraprevotella* (genus); *Adlercreutzia* (genus); *Lachnospira* (genus); *Oscillospira* (genus); *Bacteroides* (genus); *Parabacteroides* (genus); *Turicibacter* (genus); *Coprococcus* (genus); *Lactococcus* (genus); *Odoribacter* (genus); *Prevotella* (genus); *Blautia* (genus); *Ruminococcus* (genus); *Butyricimonas* (genus)	**Median (IQR)** *Paraprevotella* Control: 0.71 (0.00–4.70); endometriosis: 0.00 (0.00–1.11) (*P* < 0.001) *Adlercreutzia* Control: 6.76 (4.91–8.97); endometriosis: 5.15 (3.10–7.31) (*P* < 0.001) *Bacteroidales* Control: 0.63 (0.00–2.69); endometriosis: 0.00 (0.00–0.50) (*P* < 0.001) *Lachnospira* Control: 12.43 (11.60–13.31); endometriosis: 3.47 (1.34–4.88)–(*P* < 0.001) *Oscillospira* Control: 10.67 (9.81–11.62); endometriosis: 11.79 (10.60–12.53) (*P* < 0.001) *Coriobacteriaceae* Control: 8.24 (6.72–9.45); endometriosis: 6.95 (5.25–8.65) (*P* = 0.001) *Bacteroides* Control: 15.29 (14.25–16.45); endometriosis: 16.08 (15.14–17.26) (*P* = 0.001) *Parabacteroides* Control: 11.27 (9.98–12.47); endometriosis: 11.92 (10.95–13.20) (*P* = 0.001) *SHA98* Control: 2.63 (0.00–5.70); endometriosis: 0.00 (0.00–4.01) (*P* = 0.004) *Enterobacteriaceae* Control 3.28 (1.06–5.56); endometriosis: 4.38 (2.30–7.16) (*P* = 0,007) *Turicibacter* Control: 4.50 (2.57–6.75); endometriosis: 2.89 (0.00–5.84) (*P* = 0,008) *Coprococcus* Control: 10.31 (9.34–11.25); endometriosis: 10.81 (9.95–11.76) (*P* = 0.009)	**Alpha diversity-Shannon index (mean ± SD)** Control: 3.34 ± 0.20; endometriosis: 3.30 ± 0.20	Both alpha and beta diversities were higher in the control group (*P* < 0.05). The abundance of 12 bacteria belonging to the classes *Bacilli*, *Bacteroidia*, *Clostridia*, *Choriobacteriia*, and *Gammaproteobacter* differed significantly between patients and controls. For the authors, these findings indicate that the intestinal microbiota may be altered in patients with endometriosis

Wang et al., 2018 [29]	DNA extraction with sequencing of V5-V4 regions	*Proteobacteria* (phylum); *Firmicutes* (phylum); *Actinobacteria* (plylum); *Bacteroidetes* (phylum); *Fusobacterium* (phylum); *Terericutes* (phylum);	*Proteobacteria* Control: 67.92% Endometriosis: 72.70% *Firmicutes* Control: 16.38% Endometriosis: 20.13% *Actinobacteria* Control: 4.78% Endometriosis: 4.44%	NA	In the peritoneal fluid of patients in both groups, there was a predominance of the phyla *Proteobacteria* and *Firmicutes*, followed by *Actinobacillus*, *Bacteroidetes*, *Fusobacteria*, and *Tenericutes*, however, without significance (*P* > 0.05).

Wei et al., 2020 [30]	DNA extraction, amplification and sequencing of the V4-V5 regions of the 16S rRNA gene	*Lactobacillus* (genus); *Streptococcus* (genus); *Gardnerella* (genus); *Prevotella* (genus); *Veillonella* (genus); *Atopobium* (genus); *Veillonellaceae* (genus); *Pseudomonas* (genus); *Acinetobacter* (genus); *Vagococcus* (genus); *Comamonas* (genus)	*Lactobacillus* Control: 64.3% Endometriosis: 63.9%;	NA	From the lower to the upper reproductive tract, a significant difference in microbiota distribution was presented in the cervical mucus of patients with endometriosis (*P* < 0.05). This study also highlights the decrease of *Lactobacillus* in the vaginal flora of patients with endometriosis.

Yuan et al., 2022 [34]	Polymerase chain reaction (PCR) was performed to amplify the V4 region of the bacterial 16S rRNA gene	*Ralstonia* (genus) *Acinetobacter* (genus) *Pseudomonas* (genus) *Asticcacaulis* (genus) *Methyloversatilis* (genus)	**Peritoneal fluid–CI (confidence interval)** **(genus level)** *Ralstonia* Endometriosis: 11.15 (10.51–11.80); control: 11.47 (10.08–12.81) *Acinetobacter* Endometriosis: 2.33 (1.77–2.89); control: 2.81 (2.05–3.57) *Pseudomonas* Endometriosis: 1.53 (1.04–2.01); control: 1.75 (1.15–2.34) *Asticcacaulis* Endometriosis: 1.30 (0.95–1.65); control: 1.70 (1.29–2.11) *Methyloversatilis* Endometriosis: 0.81 (0.61–1.02); control: 1.03 (0.67–1.38)	**Alpha diversity-Shannon index–CI (confidence interval)** Endometriosis: 1.55 (1.46 –1.63) Control: 1.65 (1.59–1.68) **Simpson index–CI (confidence interval)** Endometriosis: 0.329 (0.324–0.336) Control: 0.321 (0.312 –0.334)	The microbiota diversity was similar in the peritoneal fluid of women with or without endometriosis. Ralstonia mainly dominated in the peritoneal fluid of patients in both groups (11.15%; 95% CI: 10.51–11.80% in endometriosis patients), followed by *Acinetobacter*, *Pseudomonas*, *Asticcacaulis*, and *Methyloversatilis*, with no significant difference between endometriosis patients and the control group. There were microbes with different abundance in peritoneal fluid of the two groups, and the relative abundance was less than 0.5%. *Acidovorax* (*P* = 0.01), *Devosia* (*P* = 0.03), *Methylobacterium* (*P* = 0.03), *Phascolarctobacterium* (*P* = 0.03), and *Streptococcus* (*P* = 0.04) were more abundant in the peritoneal fluid of endometriosis patients than the controls, while *Brevundimonas* (*P* = 0.01) and *Stenotrophomonas* (*P* = 0.04) were less abundant

Legend: SD: standardized deviation; NA: not available; PCR: polymerase chain reaction; LBS: lactobacillus selection agar; IQR: interquartile range; DIE: deeply infiltrating endometriosis; LVFX: levofloxacin; CI: confidence interval.

**Table 4:** Quality assessment of included studies using the Newcastle-Ottawa scale.

Author/year	Selection				Comparability	Exposure/result			Total
	1	2	3	4		1	2	3	
Case-control studies									
Akiyama et al., 2019 [21]	^∗^	^∗^	^∗^	^∗^	^∗^	^∗^	^∗^	^∗^	8
Chang et al., 2022 [33]	^∗^	^∗^	^∗^	^∗^	^∗^	^∗^	^∗^	^∗^	8
Hernandes et al., 2020 [22]	^∗^	^∗^	^∗^	^∗^	^∗^	^∗^	^∗^	^∗^	8
Huang et al., 2021 [32]	^∗^	^∗^	^∗^	^∗^	^∗^	^∗^	^∗^	^∗^	8
Khan et al., 2014 [23]	^∗^	^∗^	^∗^	^∗^	^∗^	^∗^	^1^	^∗^	8
Khan et al., 2016 [24]	^∗^	^∗^	^∗^	^∗^	^∗^	^∗^	^∗^	^∗^	8
Khan et al., 2021 [31]	^∗^	^∗^	^∗^	^∗^	^∗^	^∗^	^∗^	^∗^	8
Lee et al., 2021 [25]	^∗^	^∗^	^∗^	^∗^	^∗^	^∗^	^∗^	^∗^	8
Nabiel et al., 2020 [26]	^∗^	^∗^	^∗^	^∗^	^∗^	^∗^	^∗^	^∗^	8
Shan et al., 2021 [27]	^∗^	^∗^	^∗^	^∗^	^∗^	^∗^	^∗^	^∗^	8
Svensson et al., 2021 [28]	^∗^	^∗^	^∗^	^∗^	^∗^	^∗^	^∗^	^∗^	8
Wang et al., 2018 [29]	^∗^	^∗^	^∗^	^∗^	^∗^	^∗^	^∗^	^∗^	8
Wei et al., 2020 [30]	^∗^	^∗^	^∗^	^∗^	^∗^	^∗^	^∗^	^∗^	8
Yuang et al. 2022 [34]	^∗^	^∗^	^∗^	^∗^	^∗1^	^∗^	^∗^	^∗^	8
Cohort study									
Ata et al., 2019 [19]	—	^∗^	^∗^	^∗^	^∗^	^∗^	—	^∗^	6
Cross-sectional study									
Perrotta et al., 2020 [20]	—	^∗^	^∗^	^∗^	^∗^	^∗∗^	^∗^		7

Case-control studies: selection: (1) is the case definition adequate? (2) Representativeness of cases; (3) selection of controls; (4) definition of controls; comparability: (1) comparability of cases and controls based on design or analysis; exposure: (1) exposure verification; (2) same calculation method for cases and controls; (3) Nonresponse rate. Cohort study: selection: (1) representativeness of the exposed cohort; (2) selection of the unexposed cohort; (3) exposure verification; (4) demonstration that the result of interest was not present at the beginning of the study; comparability: (1) comparability of cohorts based on design or analysis; result: (1) result evaluation; (2) follow-up was long enough for results to occur; (3) Adequacy of cohort follow-up. Cross-sectional study: Selection: (1) representativeness of the sample; (2) sample size; (3) nonresponders; (4) verification of exposure (risk factor); comparability: (1) subjects in different outcome groups are comparable based on study design; result: (1) result evaluation; (2) statistical test;.

## Data Availability

All data used to support the findings of this study are included within the article.

## Abbreviations

CI: Confidence interval
IL: Interleukin
LPS: Lipopolysaccharide
NOS: Newcastle-Ottawa scale
OUT: Operational taxonomic units
PRISMA: Preferred reporting items for systematic reviews and meta-analyses
PROSPERO: International prospective register of ongoing systematic reviews
SMD: Standardized mean difference
TLR: Toll-like receptor
TNF: Tumor necrosis factor.
